# Confusion after spine injury: cerebral fat embolism after traumatic rupture of a Tarlov cyst: Case report

**DOI:** 10.1186/1471-227X-10-18

**Published:** 2010-08-15

**Authors:** Corina M Duja, Christophe Berna, Stéphane Kremer, Claude Géronimus, Jacques Kopferschmitt, Pascal Bilbault

**Affiliations:** 1 Division of Emergency and Intensive Care, Faculty of Medicine, Hôpital de Hautepierre, Strasbourg, France; 2 Department of Neuroradiology, Faculty of Medicine, Hôpital de Hautepierre, Strasbourg, France

## Abstract

**Background:**

Acute low back pain is a very common symptom and reason for many medical consultations. In some unusual circumstances it could be linked to a rare aetiology.

**Case presentation:**

We report a 70-year-old man with an 8-month history of left posterior thigh and leg pain who had sudden confusion after a fall from standing. It was due to cerebral fat embolism suspected by computed tomography scan, later confirmed by brain magnetic resonance imaging (MRI). A spinal MRI scan was then performed and revealed a sacral fracture which drained into an unknown perineurial cyst (Tarlov cyst). Under medical observation the patient fully recovered within three weeks.

**Conclusions:**

Sacral perineurial cysts are rare, however they remain a potential cause of lumbosacral radiculopathy.

## Background

In Emergency Care, patients admitted for low back pain with or without sciatica are common. Specific aetiology of low back pain is rarely found and does not need routinely imaging exploration [1]. But if physical examination is abnormal or pain unusually persistent, Emergency Physician should explore the lumbar spine with a CT or MRI scan. These imaging techniques can reveal herniated disk, spinal stenosis, early spinal infection or tumour. Nevertheless other rare causes can occur as in our observation where we report a sacral fracture of an unknown Tarlov cyst complicated with cerebral fat embolism.

## Case presentation

**Figure 1:**
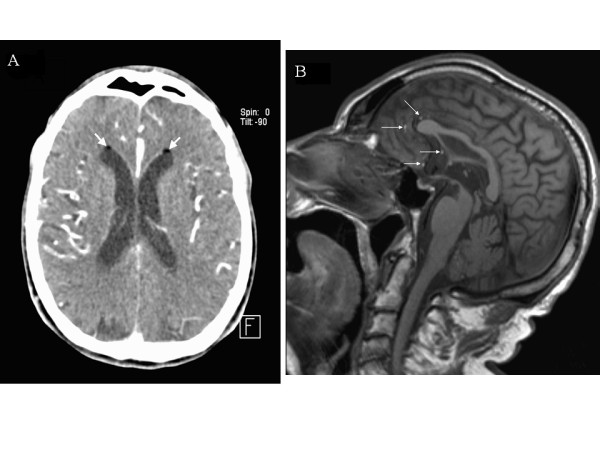
**Head CT-scan and MRI image**. A. Post contrast head CT-scan: fat droplets in the frontal horns of the lateral ventricles (white arrows). B. Sagittal *T_1_*-weighted head MR image: fat droplets disseminated in the subarachnoid spaces (white arrows).

**Figure 2:**
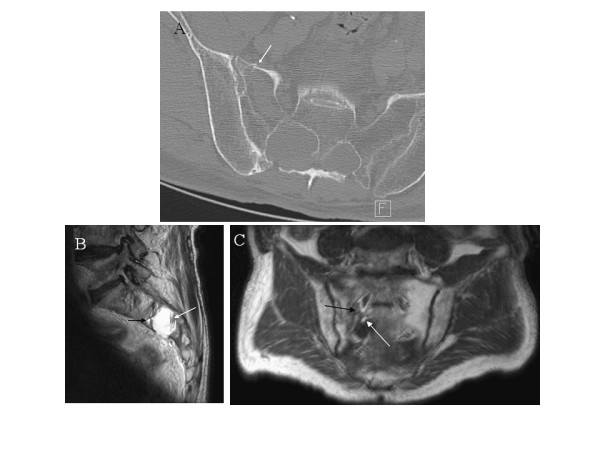
**Sacral cyst CT-scan and MRI image**. A. Axial sacral CT-scan: left sacral fracture extending to the S2 radicular cyst (presence of a contralateral cyst at the same level). B. Sagittal *T_2_*-weighted sacral MR image: S2 radicular cyst with two liquids: cerebrospinal fluid with blood sediments (white arrow) and fat droplet (black arrow). C. Coronal *T_1_*-weighted sacral MR image: left sacral fracture extending to the radicular cyst (black arrow) which contains cerebrospinal fluid and fat droplets at the bottom (white arrow).

A 70-year-old man with an 8-month history of left posterior thigh and leg pain was admitted to our Emergency Department after a fall during a gym session. He presented with a moderate pelvic and head trauma. A physical examination showed only tenderness upon palpation and percussion of the lumbar and sacral spine. Plain radiographic examinations of spine, pelvis and chest were interpreted as normal. The patient had no medical or surgical history other than essential hypertension. A few hours after admission, he became very confused and agitated. A cerebral computed tomography scan did not show either vascular lesion or cerebral contusion but fat droplets in the lateral ventricles (Figure 1A). A further investigation with CT scan of the spine revealed a fractured sacrum extending into a ruptured perineurial cyst (Figure 2A). A cerebral and spinal magnetic resonance image (MRI) scan confirmed these findings (Figures 1B, 2B-C) and we suspected that fatty bone marrow had migrated from sacral fracture to the brain in an unusual way: a dural breach at the Tarlov cyst. Surgical treatment was not carried out because of the fractured sacrum. The patient remained under medical observation and fully recovered within three weeks. Two months after discharge, the patient had no complaints and had a normal physical neurological examination.

## Discussion

Tarlov cysts were first described in 1938 as an incidental finding at autopsy of fillum terminale [2]. Then Tarlov described cases of symptomatic (low back pain) perineurial cyst and recommended their surgical removal with sacral laminectomy and excision of the cyst along the nerve root [3]. More recently, Paulsen et al [4] reported an incidence of Tarlov cysts which accounted for 1% of all back pains reported. They are more common in females [4]. The usual clinical presentations are pain in the lower back, sciatica, coccydynia or cauda equina syndrome. Usually, pain is intermittent and most frequently exacerbated by standing, walking and coughing. Tarlov's perineurial cysts were initially described in the posterior sacral or coccygeal nerve roots [3]. These cysts occur at the junction of the dorsal ganglion and the posterior nerve root and are located between the endoneurium and perineurium. They are filled with cerebrospinal fluid (CSF). The pathogenesis remains unclear: the cysts develop after congenital arachnoidal proliferations within the root sleeve or because of inflammation followed by inoculation of CSF. So the result is an obstruction of CSF flow causing cystic dilatation: CSF can enter the cyst but with restriction of its outflow. This effect has been described as a "ball valve" mechanism [5]. MRI scan is currently the imaging study of choice which reveals the cysts arising from the sacral nerve root near the dorsal root ganglion [6,7]. When cysts are symptomatic and medical treatment (analgesic and physical therapy) is unsuccessful a surgical excision is then the treatment of reference. The goal of the surgical treatment is to relieve neural compression and to stop bone erosion. There is still no consensus on the appropriate surgical indications and techniques but percutaneous drainage or microsurgical excision combined with duraplasty or plication of the cyst wall appear to be effective and safe [8,9].

Fat embolism, now called fat embolism syndrome (FES), occurs mainly after orthopaedic injuries (lower extremity trauma and intra-medullary surgery), but it has also been seen after non-trauma conditions such as closed-chest cardiac massage and acute pancreatitis[10]. Less frequently, FES was described after spontaneous or post trauma rupture of craniopharyngioma cyst [11] or after rupture of epidermoid cyst [12]. More recently Aydin et al. described, in an experimental model, that pulmonary contusion induced more cerebral fat embolism than long bone fracture and highlighted the importance of lung pathologies in the occurrence of FES [13]. The exact incidence and mortality rate are still unknown [10]. FES usually manifests as a multisystem disorder with a cascade of clinical signs such as petechial rash, deteriorating mental status, and progressive respiratory insufficiency, usually occurring within 24 hours of injury. The clinical diagnosis is based on the scale of Gurd and Wilson [14] who stated that at least two major signs or one major and four minor signs must be present (among a panel of 12 items). But for a decade the diagnosis has been based on MRI images which typically show hyperintense dot-like lesions disseminated into the brain. In diffusion-weighted MRI imaging there are multiple microembolic infarctus mimicking "starfield" pattern [15]. The treatment is on the immobilization of the fracture with supportive care. Maintaining the oxygenation of the peripheral tissues is utmost importance. The majority of cerebral fat embolism patients recover without sequelae [10].

## Conclusion

Although perineurial cysts are rare, they should be considered in the diagnosis of cerebral fat embolism after lower back injury.

## Competing interests

The authors declare that they have no competing interests.

## Authors' contributions

CMD and CB recruited the patient and helped to draft the manuscript. SK performed the images. CG and PB drafted the manuscript. JK reviewed the final version. All authors read and approved the manuscript.

## Consent

Written informed consent was obtained from the patient for publication of this case report and any accompanying images. A copy of the written consent is available for review by the Editor-in-Chief of this journal.

## Pre-publication history

The pre-publication history for this paper can be accessed here:

http://www.biomedcentral.com/1471-227X/10/18/prepub
